# Treatment of hemimasticatory spasm secondary to parry-romberg syndrome *via* partial resection of the trigeminal nerve motor branch under intraoperative neurophysiological monitoring: A case report and literature review

**DOI:** 10.3389/fsurg.2023.1146163

**Published:** 2023-04-20

**Authors:** Haitong Xu, Bin Xu, Xianjian Huang, Doudou Zhang, Xiaodong Cai

**Affiliations:** ^1^ Department of Neurosurgery, Shenzhen Second People's Hospital, The First Affiliated Hospital of Shenzhen University, Shenzhen, China; ^2^ Graduate School, Guangzhou Medical University, Guangzhou, China; ^3^ School of Medicine, Shenzhen University School of Medicine, Shenzhen, China

**Keywords:** parry-Romberg syndrome (PRS), hemimasticatory spasm, partial resection of the trigeminal nerve motor branch, masticatory muscles, spasms, intraoperative neurophysiological monitoring

## Abstract

Parry-Romberg syndrome (PRS) combined with hemimasticatory spasm (HMS) is a rare craniofacial disorder characterized by unilateral facial tissue atrophy with paroxysmal involuntary contractions of the jaw-closing muscles. Although a majority believe that this is a result of demyelination changes from the effect of the facial involvement of PRS on the trigeminal nerve motor branches, the mechanism of PRS is presently unclear. Moreover, the therapeutic effects of existing drugs that target PRS have not been satisfactory. For intolerable spasms of the masticatory muscles, botulinum toxin injection may temporarily relieve the symptoms of spasms. We report a case of HMS secondary to PRS that was treated *via* a partial resection of the trigeminal nerve motor branch under intraoperative neurophysiological monitoring.

## 1. Introduction

Parry-Romberg syndrome (PRS), also known as progressive hemifacial atrophy (PHA), is a rare acquired degenerative pathological condition. It is characterized by unilateral involvement of the facial and scalp skin, eyes, subcutaneous tissue, muscles, and bones; the rare atrophy of the trunk and limbs; and the recently discovered ipsilateral parotid atrophy, which can also accumulate bilaterally (1–5). The disease is usually self-limiting; however, its etiology is presently unclear. The main goal of PRS treatment is to halt disease progression with medications or early surgical therapy, followed by surgical correction of residual deformity (3–5).

Hemimasticatory spasm (HMS) is another rare trigeminal nerve disorder in which one or more jaw-closing muscles, such as the masseter and temporalis, are involved, causing unilateral paroxysmal contractions in the jaw-closing muscles, that prevent voluntary closing of the mouth, which may persist during sleep, and is accompanied by muscle pain, with compensatory hypertrophy changes also often seen in the muscles affected by spasms (6–11). Although electrophysiology suggests an ectopic excitation of the trigeminal nerve motor root or motor nucleus, an abnormality that is similar to the ectopic excitation of the facial nerve in hemifacial spasm (HFS), its etiology is unclear (12). In addition, unlike HFS, the presence of compression from offending blood vessels is not usually detected in general surgeries of HMS. Current HMS treatments include oral medications, botulinum toxin injections, avulsion of the peripheral trigeminal nerve motor branch, transcutaneous electrical nerve stimulation combined with ultrasound therapy, and microvascular decompression or partial resection of the trigeminal nerve motor root (6–13).

HMS secondary to PRS is extremely rare and is predominantly marked by unilateral hemifacial atrophy with involuntary spasms of the ipsilateral masticatory muscles, with only 16 reports of such cases from 1980 to 2022 (13–22). In addition, cases that have been reported to date involved non-surgical interventions, such as medications and botulinum toxin. We report a case of partial resection of the trigeminal nerve motor branch in the treatment of PRS combined with HMS.

## 2. Case report

### 2.1. Patient's clinical information

**Figure 1:**
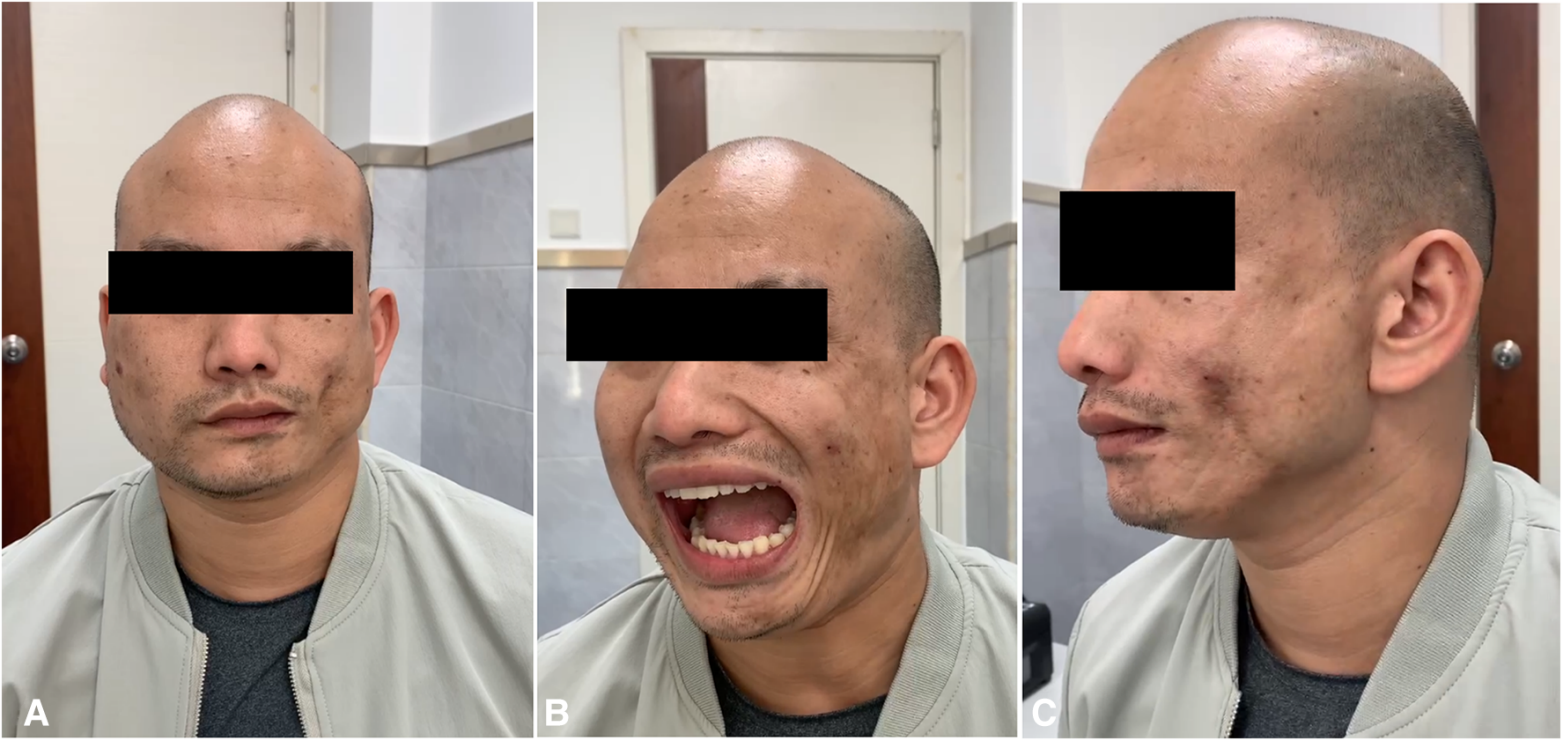
(**A–C**). Severe hemifacial atrophy on the left side and hypertrophy of the left masseter muscle.

**Figure 2:**
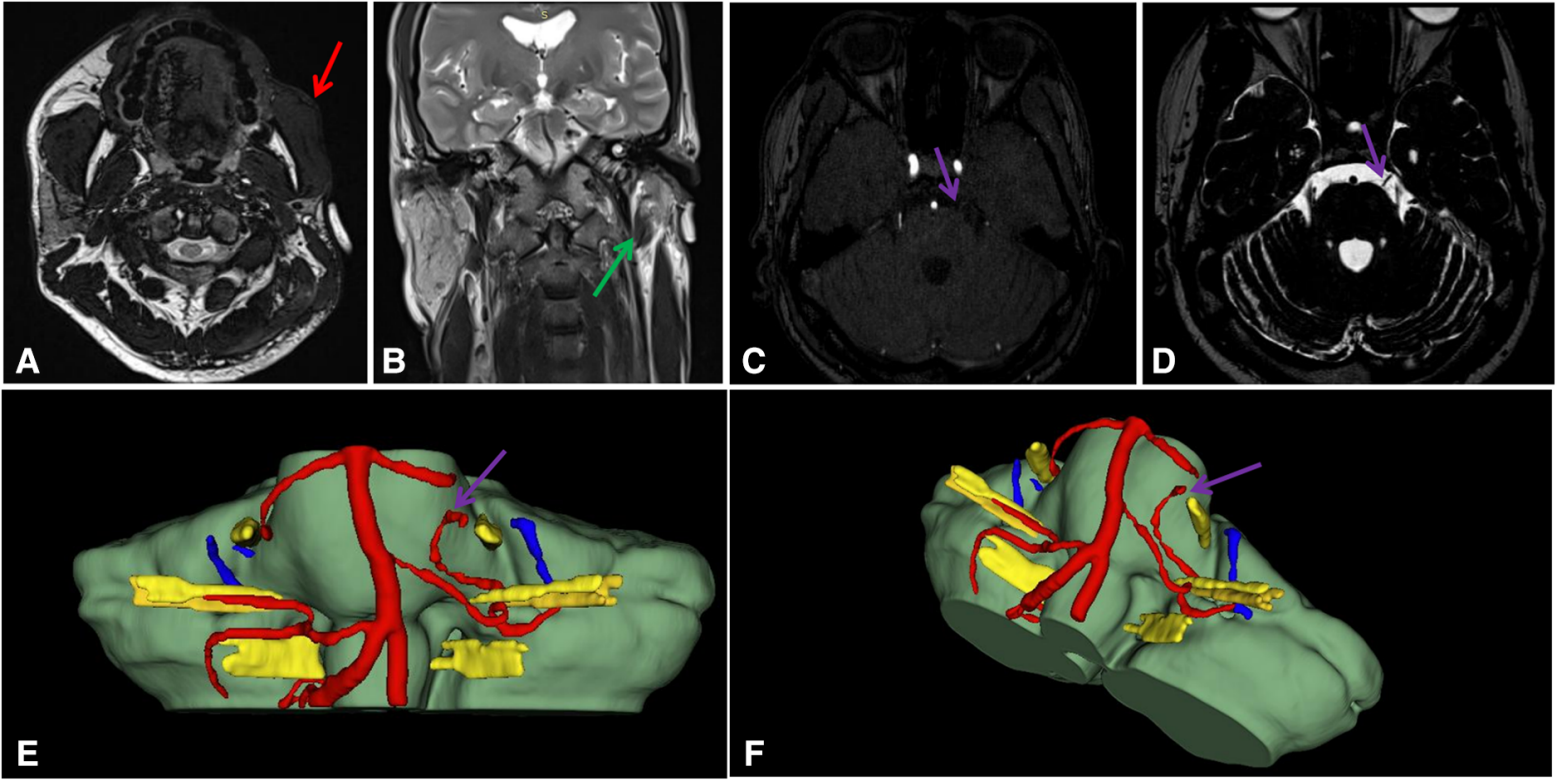
(**A–F**). Images and 3D reconstruction. (**A**). Reduction in the subcutaneous fat of the left cheek(Red arrow); (**B**). Left parotid gland atropy (Green arrow); (**C**). MRI-3D-TOF; (**D**). MRI-T2; (**E,F**). 3D reconstruction. A tiny blood vessel runs ventral to the CN V(Purple arrow).

A 29-year-old man presented with progressive worsening of atrophy of the left facial muscles, which first occurred 8 years ago. Before October, he had paroxysmal involuntary twitching of the left masticatory muscles accompanied by pain. The attack happened dozens of times daily, with each attack lasting for 3–5 s before spontaneously resolving. During the periods between the attacks, the patient was free from these symptoms. However, he experienced cold and tension that worsened with emotional aggravation, and mouth-opening movements, such as chewing, were affected. The patient had no facial numbness, dysphagia, headache, dizziness, nausea, vomiting, unsteady gait, or sore throat. His condition did not improve with oral medications. Physical examination: There was involuntary twitching and hypertrophy of the left masseter muscle and temporalis muscle. There was evidence of facial atrophy in the left corner of the mouth, and the subcutaneous fat on the left side was relatively poor compared to that on the right side, with no other abnormalities found (Figures 1A–C). No notable abnormalities were detected from the routine blood tests, biochemical tests, electrocardiogram, and chest x-rays. Carotid artery Doppler ultrasound revealed uneven thickening of the intima-media of the left carotid artery. Head CT and three-dimensional (3D) reconstruction showed an intact skull bone, and no abnormal intracranial density foci or space-occupying lesion was seen. Oral cavity MRI showed thicker left masticatory muscles compared to the contralateral side, a significant reduction in the subcutaneous fat of the left cheek, and a significantly smaller left parotid gland compared to the contralateral side. (Figures 2A, B). MRI-3D-TOF and MRI-T2 sequences showed that the morphology of both sides of the trigeminal nerve were normal, and no offending blood vessels were found (Figures 2C, D). Reconstruction by the 3D-Slicer software (Figures 2E, F). Electromyography (EMG) showed synchronous bursts of motor unit activity in the left masticatory muscles during spasms, which were most predominant in the temporalis muscle. No abnormality was detected in the conduction velocity of the motor nerve. The patient was diagnosed with left PRS combined with HMS, and a decision was made to carry out a partial resection of the left trigeminal nerve motor root under intraoperative neurophysiological monitoring.

### 2.2. Surgical procedure

**Figure 3:**
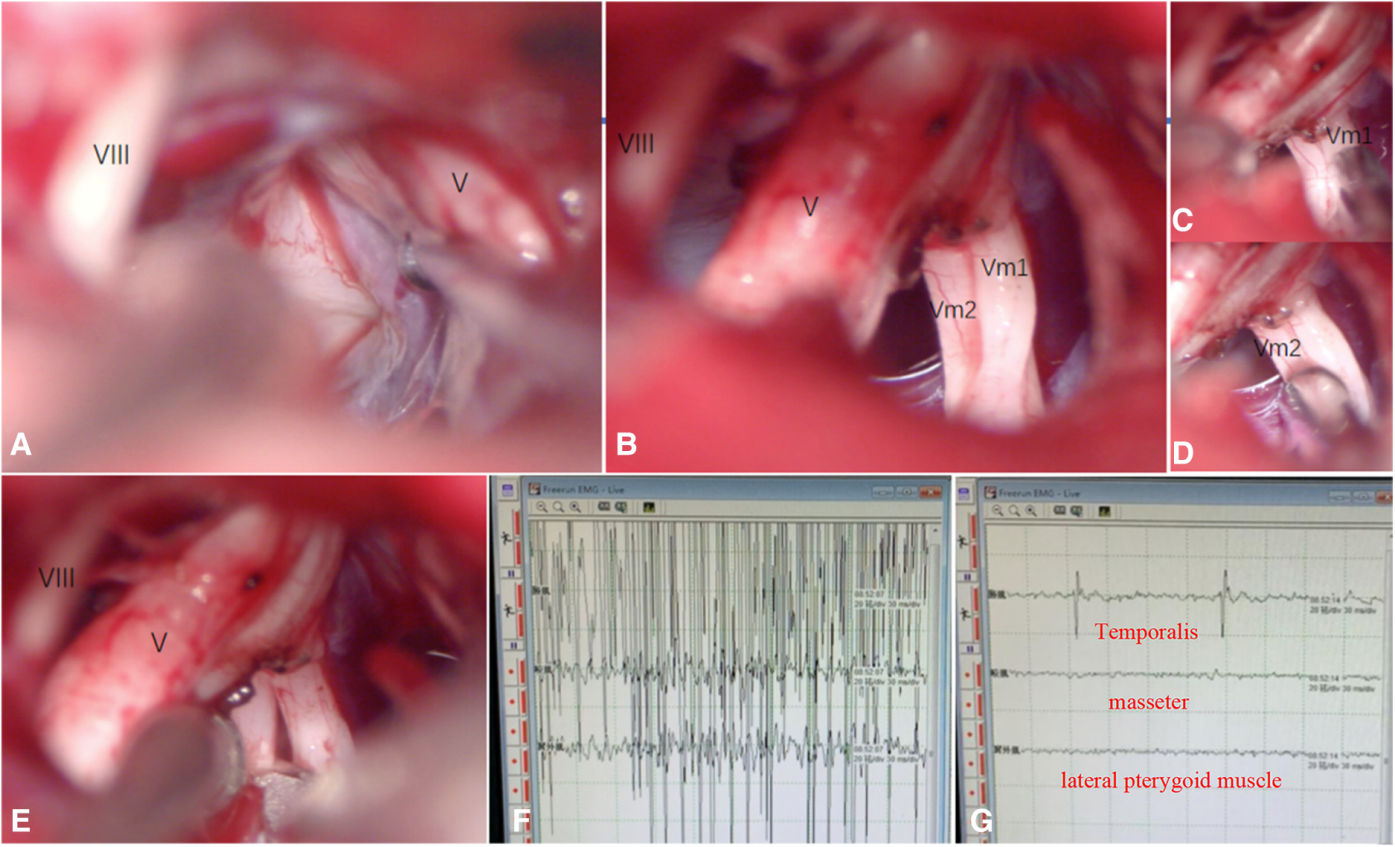
(**A–G**). Intraoperative Situation. (**C**). The masseter muscle responds to stimulation of Vm1; (**D**). The lateral pterygoid muscle responds to stimulation of Vm2; (**E**). Vm1 and Vm2 are cut off by 50%, respectively; (**F**). Preoperative EMG: Abnormal discharge was mainly in the temporal muscle (masticatory muscle spasm); (**G**). Postoperative EMG: After selective resection of the motor branch, the abnormal discharge disappeared, and masseter contraction was still observed.

**Figure 4:**
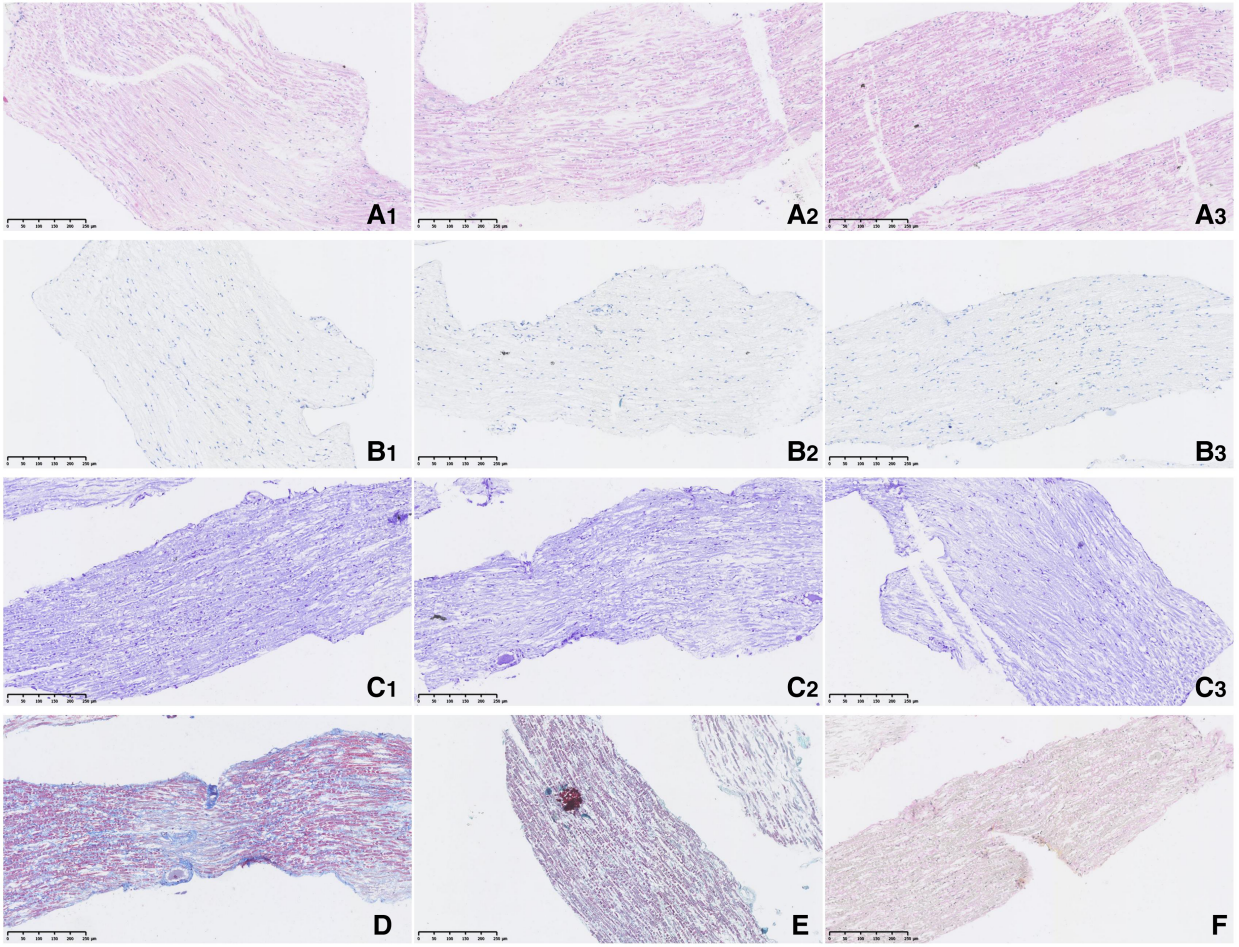
Pathological morphology of nerve tissue. (**A**). *Hematoxylin Eosin* (A1, A2, A3 × 200); (**B**). Toluidine Blue (B1, B2, B3 × 200); (**C**). *Methyl Violet* (C1, C2, C3 × 200); (**D**). Massion ( × 200); (**E**). MGT ( × 200); (**F**). Van Gieson ( × 200).

The surgery was performed under general anesthesia with tracheal intubation and in the right lateral position. Needle electrodes were inserted into the patient's left temporalis muscle, masseter muscle, and external pterygoid muscle. Abnormal discharges of varying degrees in the aforementioned muscles could be recorded during spasms of the masticatory muscles, which was most notable in the temporalis muscle. When spasms were absent, no abnormal discharges were observed, and routine neurophysiological monitoring was carried out. Conventional retrosigmoid craniotomy was performed, with a longitudinal incision of approximately 5 cm in length behind the ear and inside the hairline. A retrosigmoid craniectomy of 3 cm in diameter was created, the dura was opened, and the entire course of the trigeminal nerve root was exposed after the cerebrospinal fluid was slowly released and the surrounding arachnoidal membrane were dissected. The sensory root was separated from the motor root in the root entry zone (REZ) of the left trigeminal nerve. The trigeminal nerve motor root could be seen to be divided into two branches, with a communicating branch in the middle (Figures 3A, B). The monopolar stimulation technique was used to stimulate the cephalic and caudal branches of the trigeminal nerve motor root, where the individual EMG responses of the masticatory muscles could be recorded (Figures 3C, D), and the contraction of the temporalis and masseter muscles could be observed. 50% of the cephalic and caudal branches of the trigeminal nerve motor root were resected, along with the communicating branch. The abnormal discharges in the left masticatory muscles of the patient completely disappeared, although contractions of the temporalis and masseter muscles continued to be present (Figures 4A, B). The portion of resected nerve tissue of the trigeminal nerve motor root was sent for pathological examination during the surgery (Table 1). There was no abnormality in the brainstem auditory evoked potentials during the surgery. The dura was repaired, followed by the skull defect, and the incision was gradually sutured in layers.

## 3. Results

Following surgery, the spasms of the left masticatory muscles disappeared, occlusion was normal, and the pain was absent from the masticatory muscles. The patient was satisfied with the treatment outcome. Physical examination: The left masticatory muscles appeared to be slightly weaker than that on the right side. Other cranial nerve examinations did not show any significant abnormalities. No recurrence occurred at 3, 6, and 12 months of follow-up after surgery. However, at 14 months, there was a recurrence of spasms of the left masticatory muscles, and a mild degree of atrophy in both the temporalis muscle and masseter muscles, while no occlusal disorder was present. The self-reported symptoms improved by approximately 50% compared to before the surgery.

## 4. Discussion

PRS is an extremely rare, acquired disorder of which etiology remains unclear. It may be associated with trauma, viral infections, endocrine disorders, genetic and autoimmune conditions, neurovasculitis, lipid metabolism disorders, nutritional disorders, or hyperactivity of the sympathetic nervous system (1–5). At present, the effects of PRS treatments are uncertain, and the methods that can be used include methotrexate (MTX), steroid hormones, ultraviolet A (UVA), psoralen plus ultraviolet A (PUVA), other related immune agents or early multiple autologous fat grafting, which are able to delay or achieve early stabilization of active PRS to a certain degree (3–5).

HMS is a rare trigeminal nerve disorder that manifests as involuntary spasms of the masticatory muscles resulting in compensatory hypertrophy changes, along with pain. Of the currently available treatments, most experts prefer BTX-A injection. In uncomplicated HMS, some research has found electrophysiologically similar potentials to the facial nerve of hemifacial spasm (12–18, 20). In recent years, an increasing number of experts concur with the neurovascular conflict theory and have attempted to apply simple microvascular decompression to the treatment of HMS, all of which have achieved a certain level of therapeutic effects (6–8, 10). Wu et al. reported on the surgical treatment of 10 HMS cases, and the findings showed that treatments with pure microvascular decompression or partial or complete resection of the trigeminal nerve motor branch were not all effective and did not prevent recurrences of spasms. Hence, the etiology of HMS remains unclear, and the possibility of a joint influence from both HMS and central factors cannot be ruled out (10).

PRS and HMS are both rare cases on their own, with HMS secondary to PRS being even rarer, thus only 16 cases have been reported to date worldwide (13–22), of which all patients were treated with medications and botulinum toxin without surgical intervention. In the review of 55 patients with HMS from 1980 to 2017 by Divya M et al., approximately 25% of patients were found to have an etiology that was related to PRS (9). Experts believe that the compression or focal demyelination of the trigeminal nerve branches may be caused by anatomical changes in the deep subcutaneous tissue due to PRS (6–12, 21–22).

In the present case, the patient had no previous history of trauma or underlying conditions and developed ipsilateral typical masticatory muscle spasm after 8 years of left facial atrophy. Preoperative oral MRI and EMG showed abnormalities, and diagnoses of PRS and HMS diagnoses were established. We believe that the HMS was secondary to PRS. Preoperative imaging and intraoperative findings revealed that the morphology of the left trigeminal nerve was normal, without the presence of vascular compression. In this case, we believe that vascular compression was not the cause of HMS secondary to PRS. With neurophysiological monitoring, we accurately located the trigeminal nerve motor root and performed a partial resection of the trigeminal nerve motor root. During the surgery, we first resected 50% of the cephalic and caudal branches of the motor root, followed by the resection of the communicating branch between the two branches. Since the abnormal discharges in the muscles completely disappeared during spasms of the left masticatory muscles at that point, we did not completely resect the motor root to avoid dysfunction of the masticatory muscles. Wu et al. reported of 3 HMS cases who underwent resection of the trigeminal nerve motor root, where the spasms disappeared following surgery, and there was no recurrence at 5 years of follow-up in two cases and no recurrence at 2 years of follow-up in one case; the patients only had mild atrophy of the masticatory muscles, and mouth opening was not restricted (10). Our patient only had mild atrophy of the temporalis and masseter muscles at 14 months after surgery, without any occlusal disorder.

As with any rhizotomy, the neurosurgeon faces the challenge of eliminating symptoms and reducing recurrence while avoiding severe neurological impairment. Unfortunately, so far, there is still no objective and effective method to guide the appropriate cut-off ratio. We also tried to investigate the etiology of HMS secondary to PRS. The patient's routine postoperative neuropathological examination and special staining showed that the nerve tissue was normal. Owing to the small number of specimens and the specialized nature of neuropathology, we were unable to determine whether demyelination changes occurred in the cisternal segment of the trigeminal nerve motor root. There is a need to further explore the etiology of the two diseases and the relationship between the two.

## 5. Conclusion

Partial resection of the trigeminal nerve motor branch under intraoperative neurophysiological intraoperative monitoring in cases of HMS secondary to PRS can achieve very good therapeutic effects in the short term, and it needs to be performed with caution.

**Table 1:** Review of 16 cases with PRS accompanied with HMS.

Authors	Age/Sex	Affected muscles	Treatment for HMS	Effect	Follow up spasticity
Kaufman (1980)	25/female	Left masseter	–	–	–
Lapresle (1982)	15/female	Right masseter	–	–	–
Thompson et al. (1986)	31/female	Right masseter	Myotomy	–	–
Parisi et al. (1987)	39/female	Right masseter	Carbamazepine	Remission of spasm	–
Cruccu et al. (1994)	20/male	Left temporalis	Carbamazepine	Remission of spasm	–
	50/female	Right masseter and temporalis	Carbamazepine、Injection of BTX-A	Moderate remission of spasm	The symptoms did not relieve for 5 months
Kim et al. (1994)	44/male	Right masseter	BTX	–	–
Ebersbach et al. (1995)	36/male	Left masseter and temporalis	Injection of BTX-A	Marked remission of spasm	There was no recurrence in 4 months
	30/female	Right masseter and temporalis	Injection of BTX-A	Marked remission of spasm	There was no recurrence in 4 months
Bilen et al. (1999)	48/female	–	Phenytoin	Remission of spasm	–
Kim et al. (2000)	37/female	Right masseter	Injection of BTX-A	Marked remission of spasm	There was no recurrence in 3 months
Wei-Feng Z (2011)	57/female	Right side	BTX	–	–
Panda et al. (2014)	35/female	Left masseter and temporalis	Carbamazepine	Marked remission of spasm	–
Kim et al. (2015)	27/female	Right masseter	Trigger point injection with dexamethasone and lidocaine、Injection of BTX-A、colchicine and eperisone.(Treatment for HMS—Orthodontic surgery)	Marked remission of spasm	Intermittent seizures within 5 months; After 3 years of follow-up, the soft tissue facial asymmetry remained
Baduni (2015)	19/male	Left masseter and temporalis	ultrasound therapy and TENS	Marked remission of spasm	–
Chen GC et al. (2020)	34/male	Left masseter	Phenytoin、Injection of BTX-A	Marked remission of spasm	There was no recurrence within 10 months

PRS, Parry-Romberg syndrome; HMS, hemimasticatory spasm; BTX-A, botulinum toxin type A; TENS,Transcutaneous electric nerve stimulation.

## Data Availability

The original contributions presented in the study are included in the article/Supplementary Material, further inquiries can be directed to the corresponding authors.
